# Supplementary material for: Comparative multi-omic analysis reveals conserved and derived mechanisms of fin and limb regeneration
Source: Nat Commun. 2026 Jan 22;17:1922. doi: 10.1038/s41467-026-68801-w (PMC12923738; doi:10.1038/s41467-026-68801-w)
Supplement: Supplementary file 1 — Supplementary Information [file 41467_2026_68801_MOESM1_ESM.pdf]

Supplementary Information

Comparative multi-omic analysis reveals conserved and derived mechanisms of fin and limb regeneration

Josane F. Sousa[‡], Gabriela Lima[‡], Louise Perez, Hannah Schof and Igor Schneider*.
Department of Biological Sciences, Louisiana State University, Baton Rouge, LA, 70803.

*Corresponding author: Igor Schneider.

Email: igors@lsu.edu

[‡]These authors contributed equally

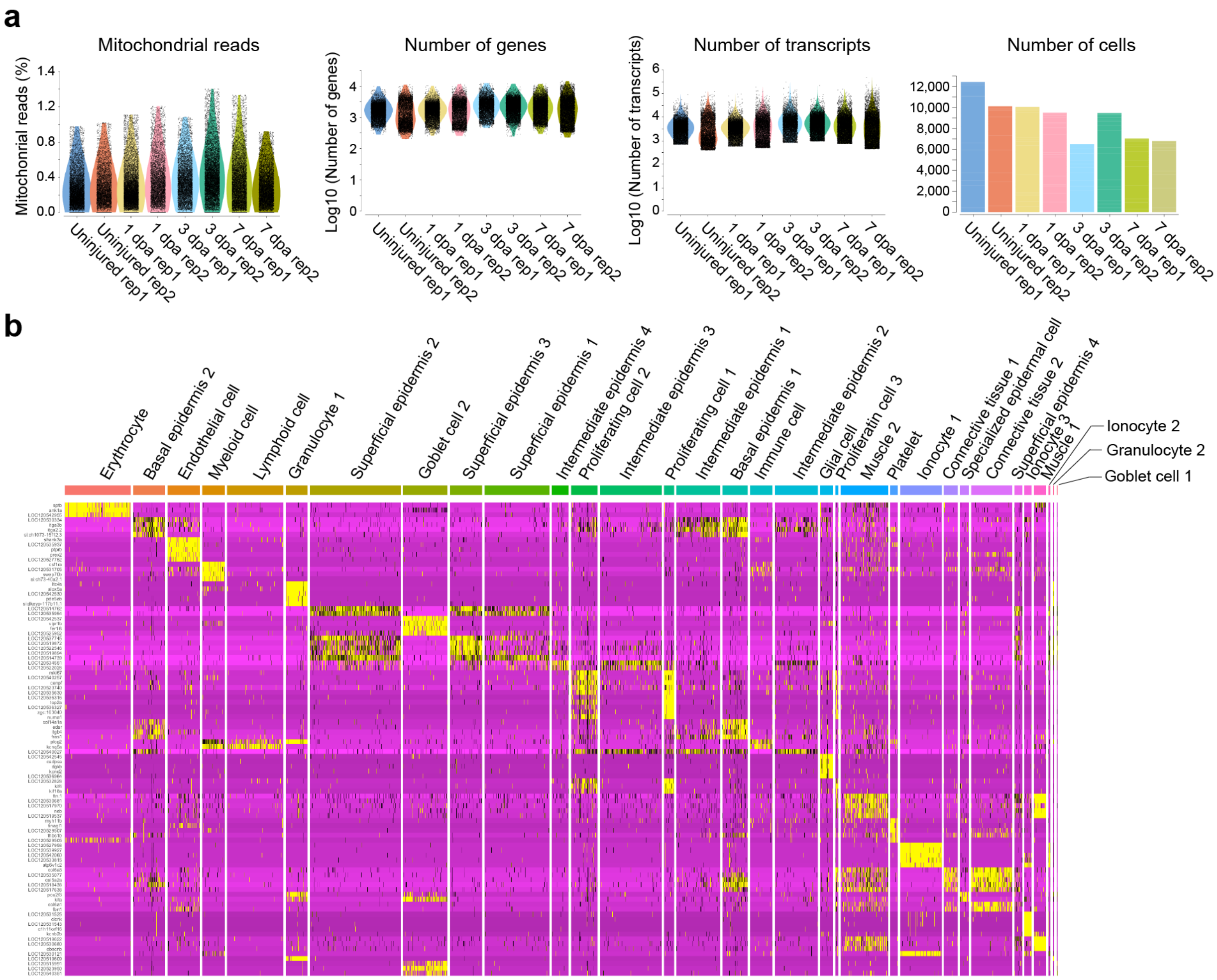

Supplementary Fig. 1. *Polypterus* fin snRNA-seq quality control metrics and marker gene expression across different cell clusters. a Percentage of mitochondrial reads, number of genes, transcripts and cells (nuclei) across samples. **b** Heatmap depicting marker gene expression across the different cell clusters identified.

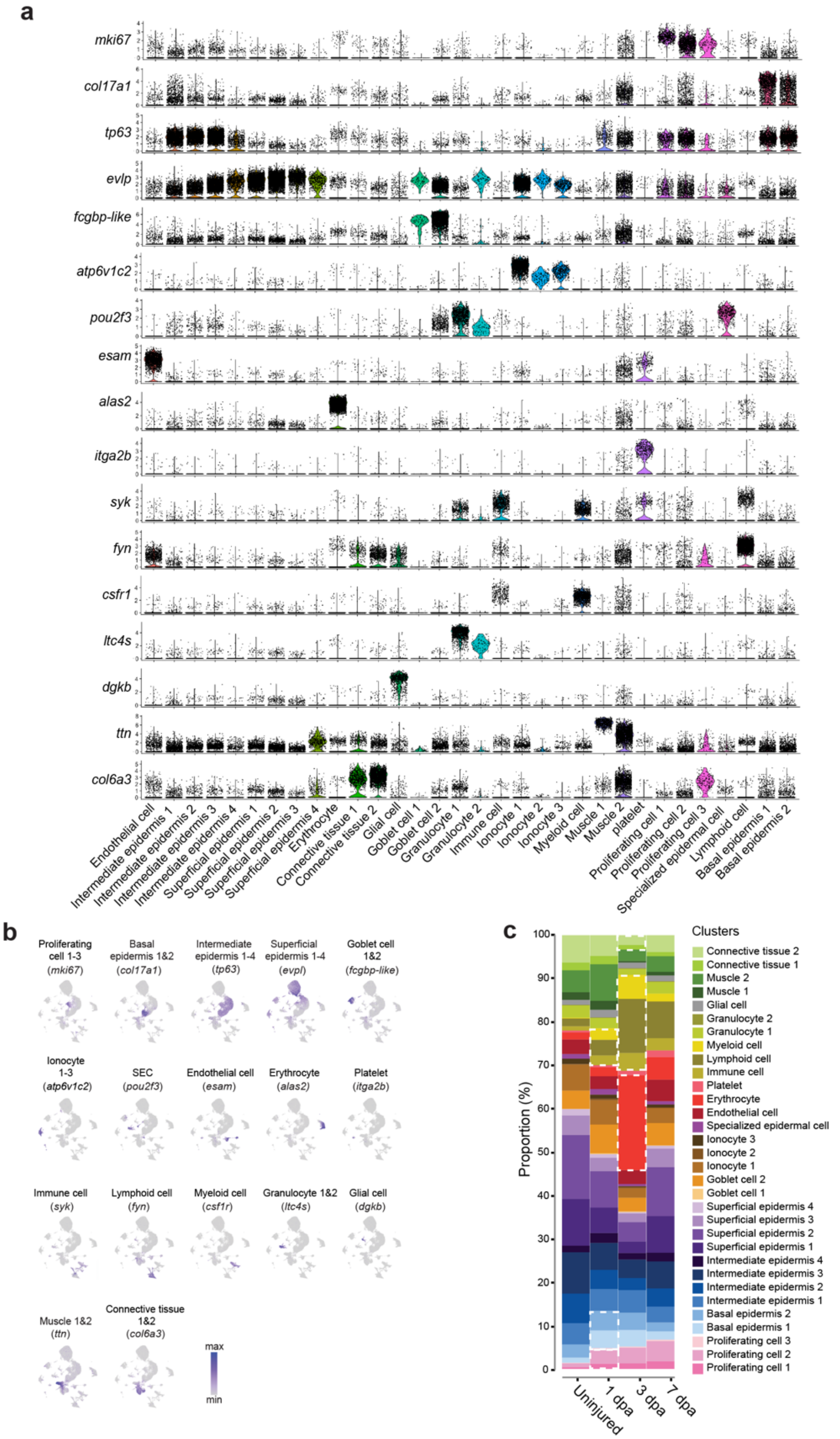

Supplementary Fig. 2. Marker gene expression of major cell populations across all stages and cell population dynamics during *Polypterus* fin regeneration. a Violin plots of select markers of major cell populations identified in all stages combined. **b** UMAP plots of representative gene markers of major cell types shown in (**a**). **c** Frequency plot showing proportions of cell clusters across regeneration stages; dashed white boxes highlight most dynamic clusters.

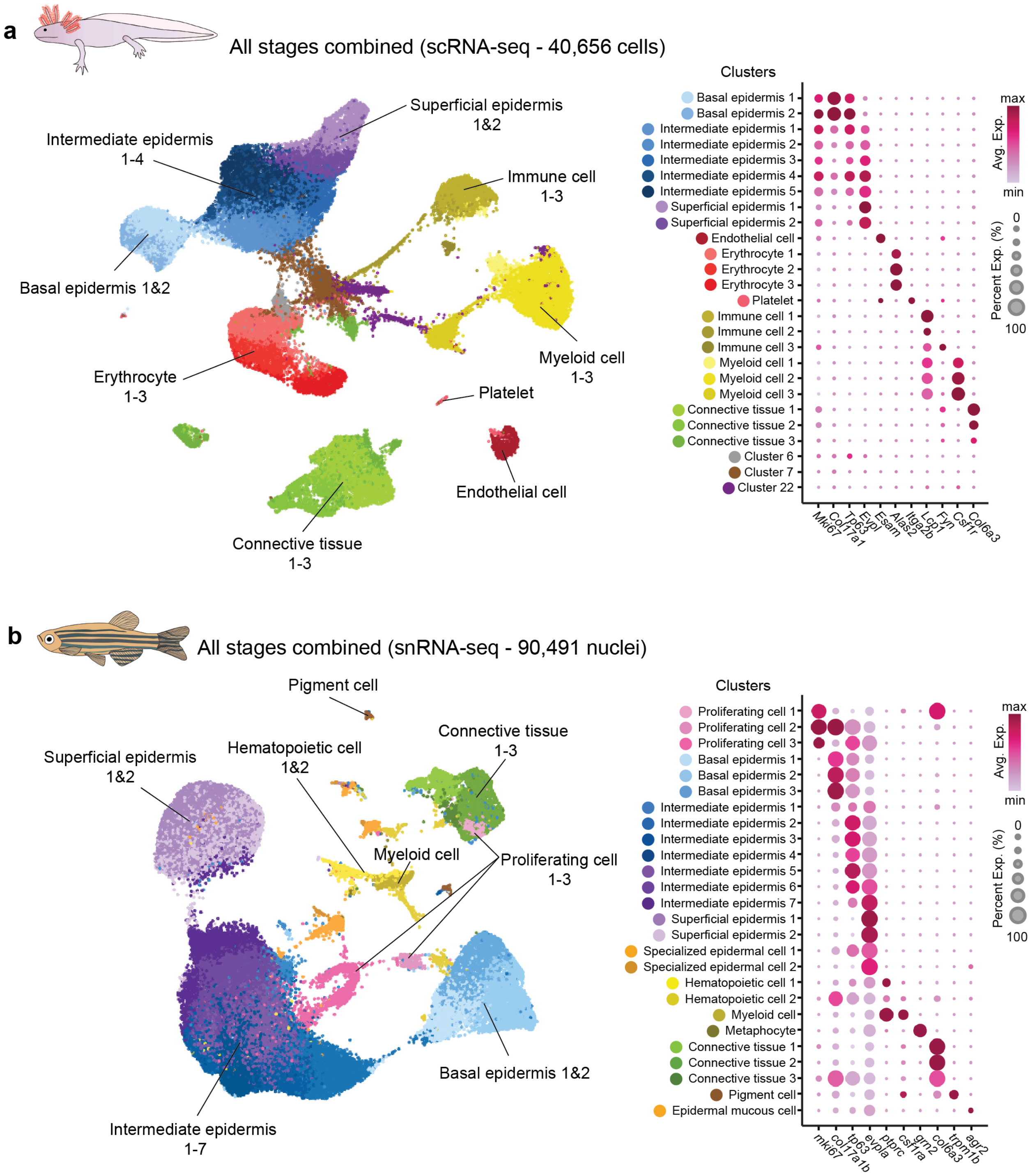

Supplementary Fig. 3. Cellular diversity of axolotl limb and zebrafish caudal fin regeneration based on reanalysis of publicly available sc/snRNA-seq datasets. a UMAP plot of axolotl limb regeneration scRNA-seq dataset[15] from all stages combined (uninjured, 3 dpa, 7 dpa, 14 dpa and 22 dpa) identified 26 distinct clusters; Dot plot of representative gene markers of major cell types identified in the axolotl dataset. **b** UMAP plot of zebrafish caudal fin regeneration snRNA-seq dataset[8] from all stages combined (uninjured, 1 dpa, 2 dpa, 4 dpa, and 6 dpa); Dot plot of representative gene markers of major cell types identified in the zebrafish dataset.

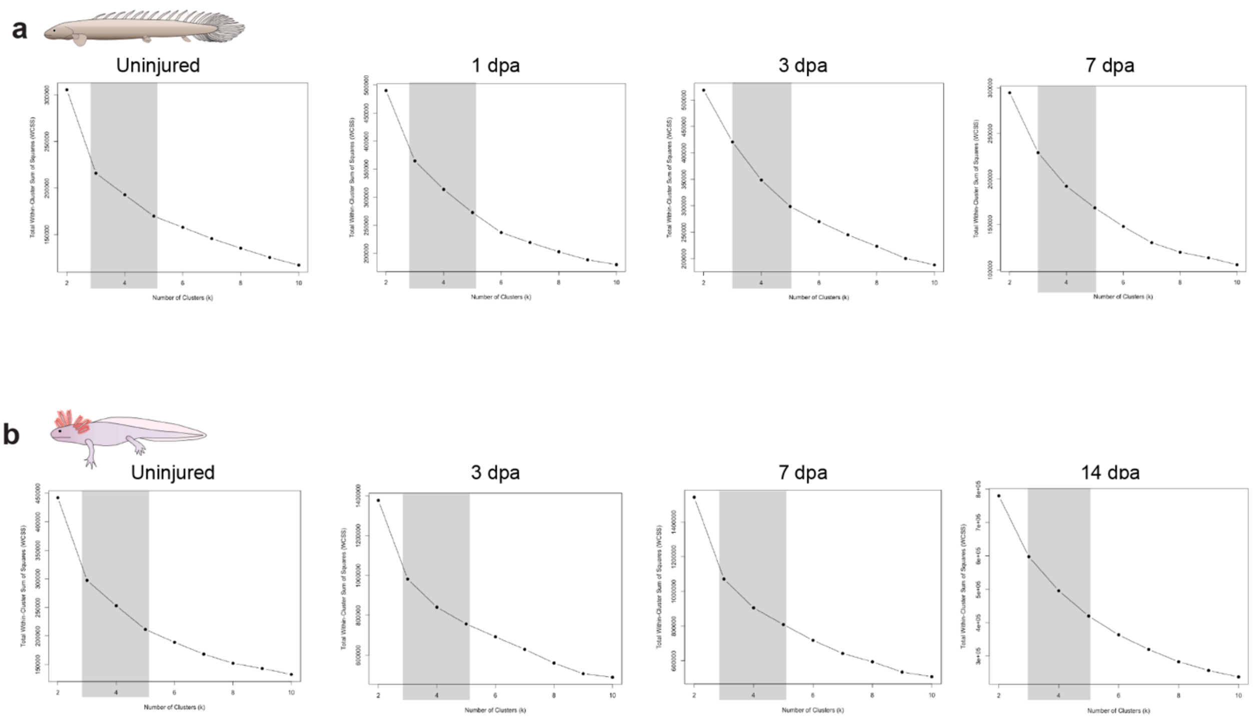

Supplementary Fig. 4. Elbow plots used to determine optimal clustering resolution for spatial transcriptomics datasets. a, b Elbow plots showing the total within-cluster sum of squares (WCSS) across k = 2-10 clusters for *Polypterus* fin (**a**) and axolotl limb (**b**) at the indicated time points. The shaded areas denote inflection point of each curve where additional clusters yield diminishing returns in model fit. Selected clustering range (k = 4-5) used for subsequent analyses.

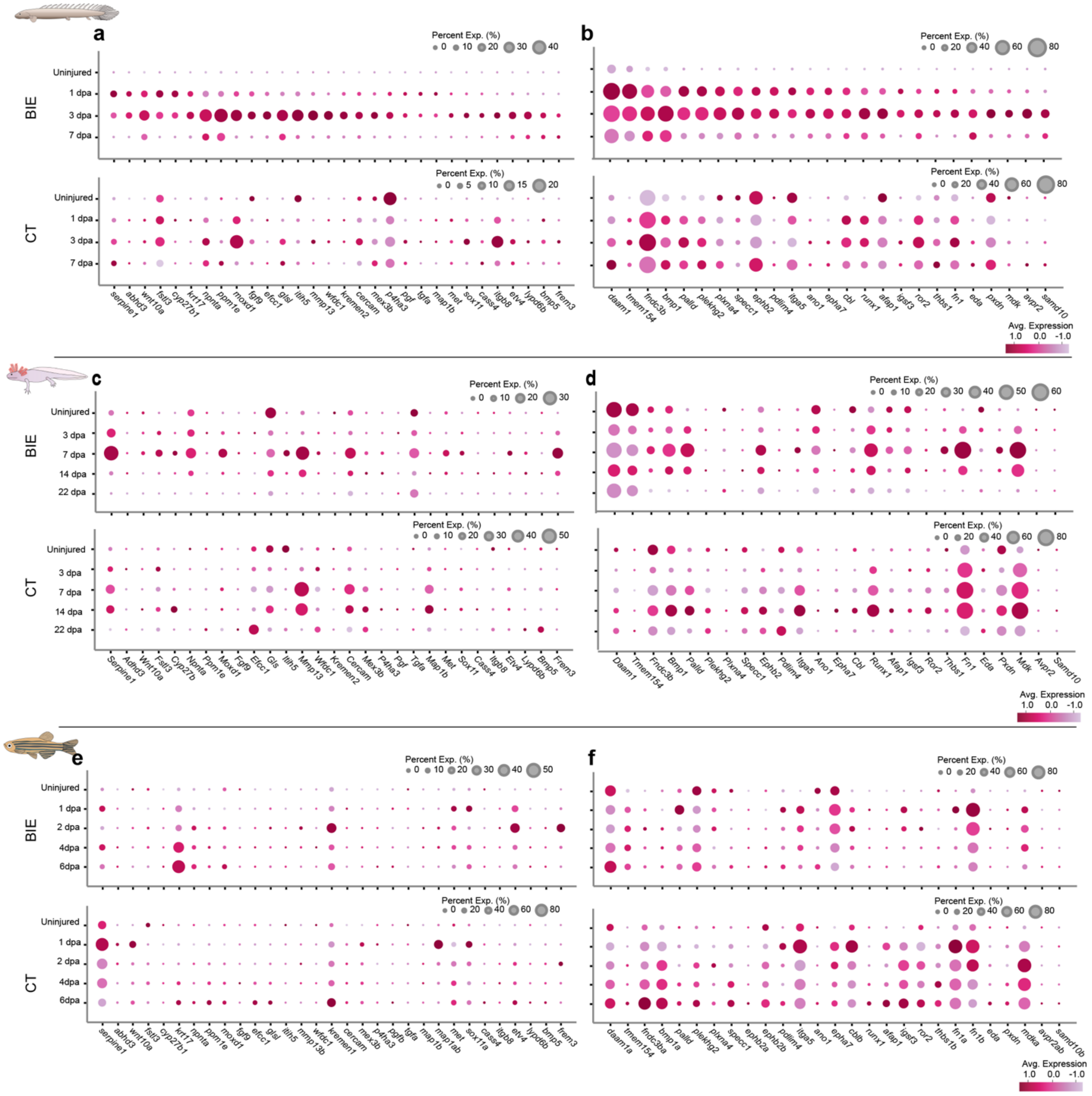

Supplementary Fig. 5. *Polypterus* wound epidermis upregulated genes and their expression pattern during axolotl limb and zebrafish caudal fin regeneration. a, b Dot plots based on snRNA-seq data showing the expression patterns of two groups of genes found upregulated in the basal and intermediate epidermis (BIE) of *Polypterus* regenerating fins: Top 30 upregulated genes with expression more restricted to the epidermal compartment, and expressed only in small populations of cells in other compartments such as the connective tissue (CT) (**a**). Top 25 upregulated genes with broader expression in the epidermis as well in other cell compartments such as the connective tissue (**b**). **c-f** Several of those genes are also upregulated during axolotl limb (**c** and **d**) or zebrafish caudal fin (**e** and **f**) regeneration.

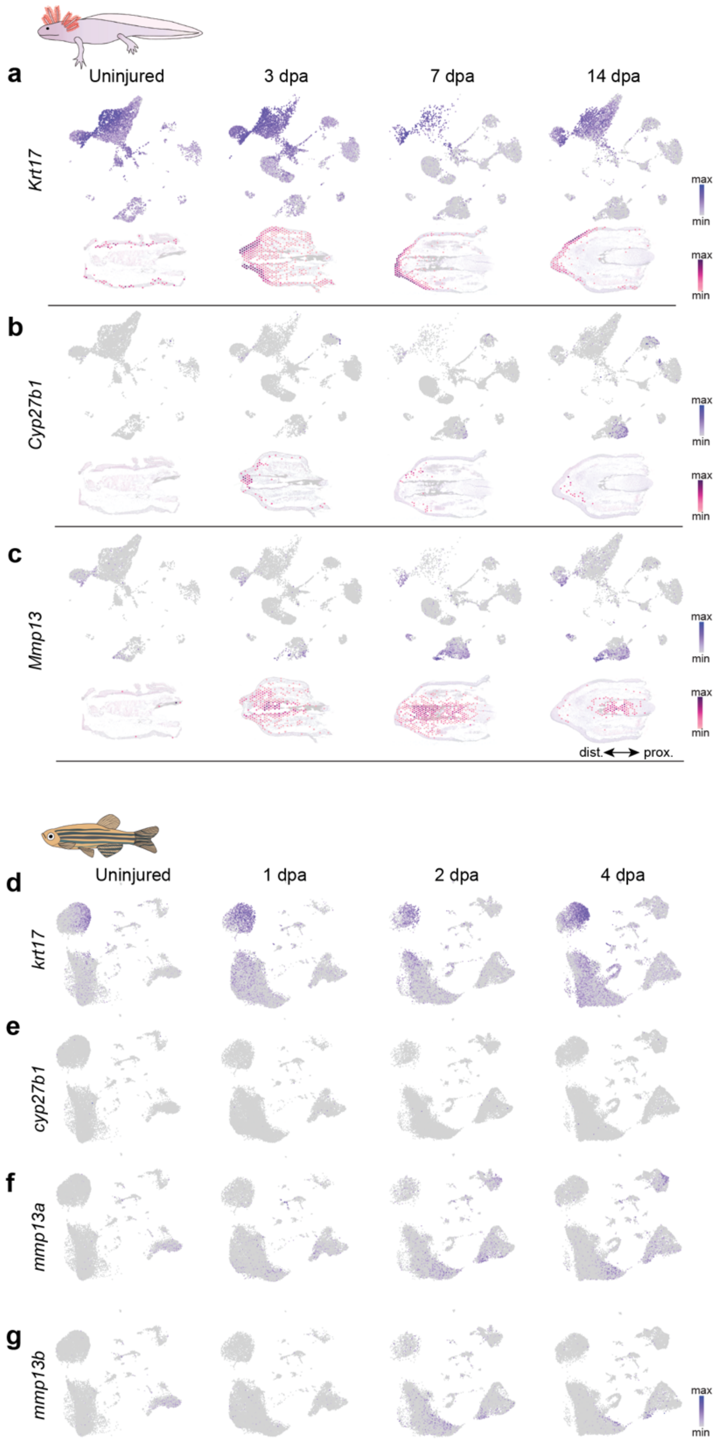

Supplementary Fig. 6. Expression of select *Polypterus* wound epidermis markers during axolotl limb and zebrafish caudal fin regeneration. a-c UMAP plots (top) and spatial RNA-seq (bottom) of axolotl *Krt17* (**a**), *Cyp27b1* (**b**), and *Mmp13* (**c**) in the uninjured tissue and across regeneration stages. **d-g** UMAP plots of zebrafish *krt17* (**d**), *cyp27b1* (**e**), *mmp13a* (**f**), and *mmp13b* (**g**). Proximal (prox.) distal (dist.) axis indicated in **c.**

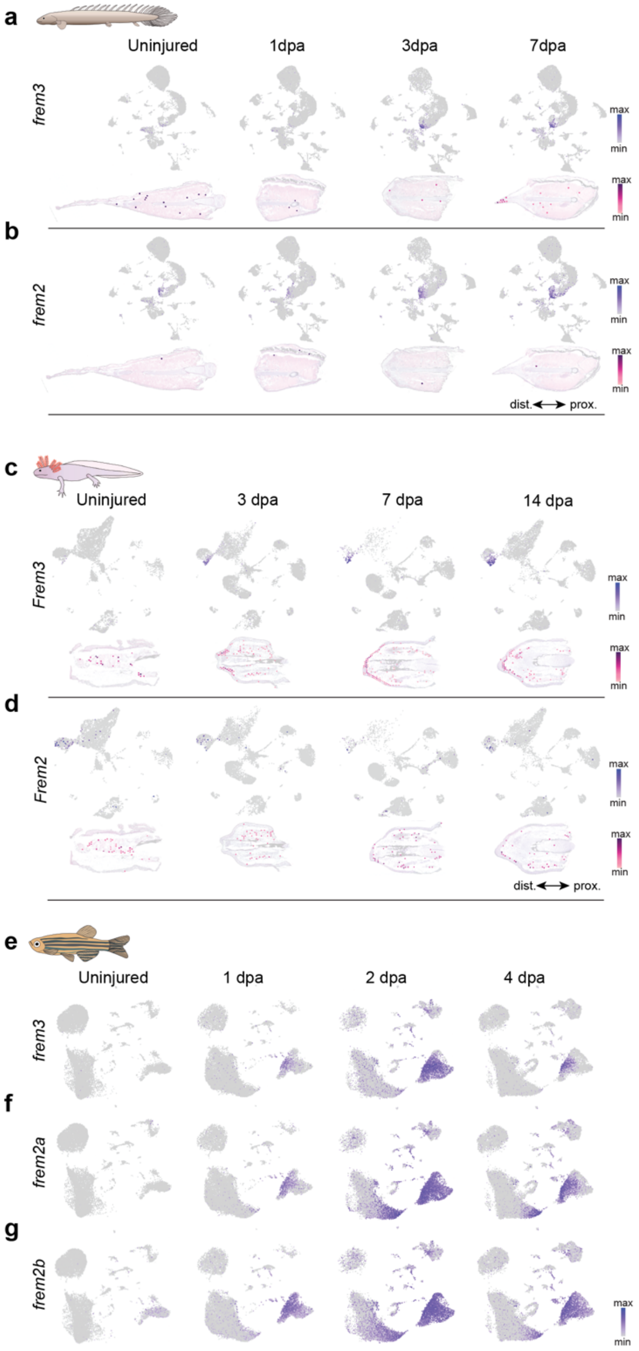

Supplementary Fig. 7. Expression of *Polypterus*, axolotl and zebrafish *frem2/3* orthologs during limb and fin regeneration. a, b UMAP plots (top) and spatial RNA-seq (bottom) showing expression of *frem3* (**a**) and *frem2* (**b**) in the *Polypterus* uninjured fin and during regeneration stages. **c, d** UMAP plots (top) and spatial RNA-seq (bottom) showing expression of *Frem3* (**c**) and *Frem2* (**d**) in the axolotl uninjured limb and during regeneration stages. **e-g** UMAP plots showing expression of *frem3* (**e**), *frem2a* (**f**), and *frem2b* (**g**) in the uninjured zebrafish caudal fin and during regeneration stages. Proximal (prox.) distal (dist.) axis indicated in **b** and **d.**

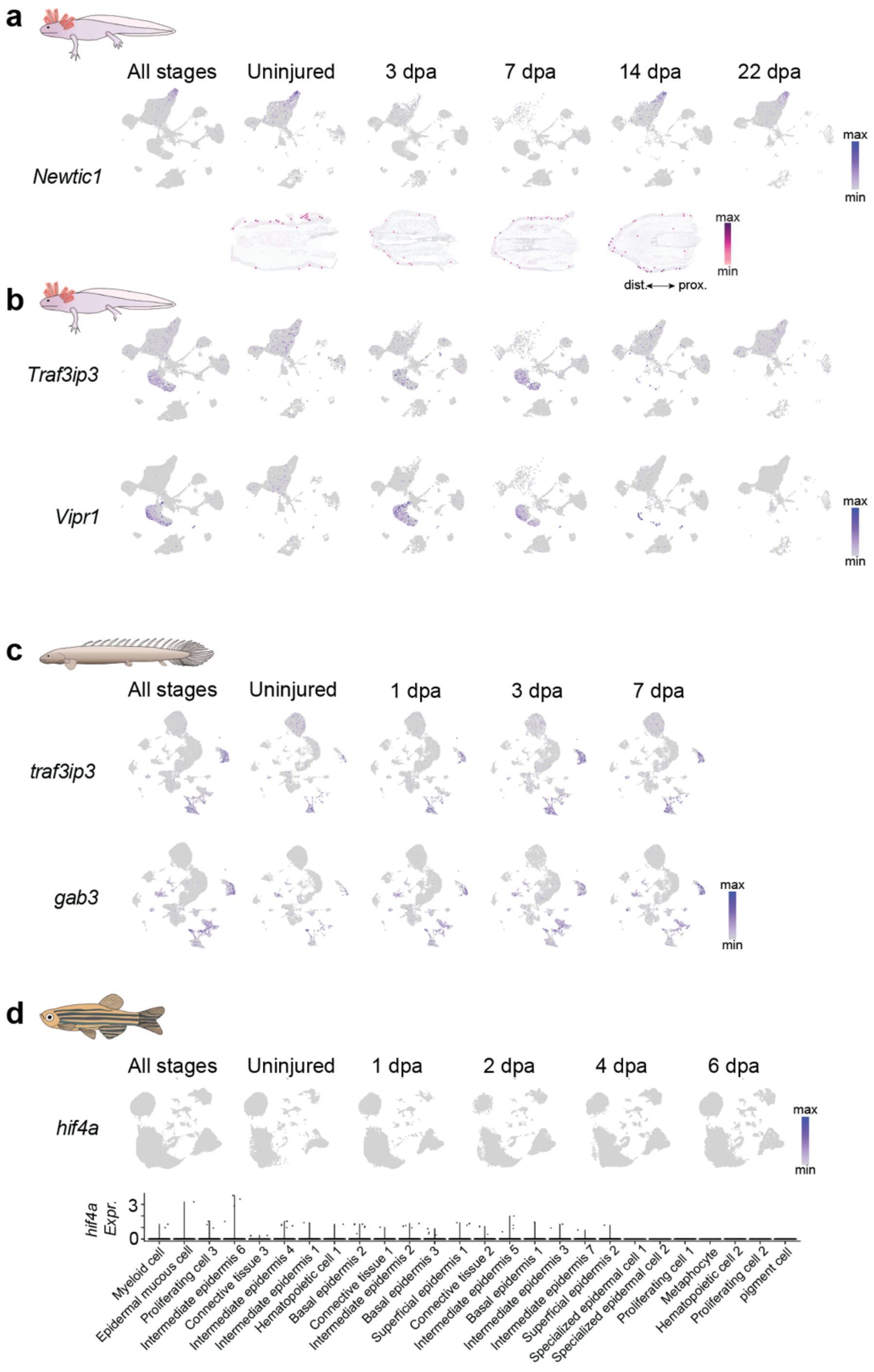

Supplementary Fig. 8. Expression patterns of conserved and species-specific atypical erythroid genes across cell clusters. a UMAP plots (top) and spatial RNA-seq (bottom) expression of *Newtic1* in the uninjured axolotl limb and during regeneration. **b** UMAP plots of *Traf3ip3* and *Vipr1* in the uninjured axolotl limb and during regeneration. **c** UMAP plots showing expression of *traf3ip3* and *gab3* in the uninjured *Polypterus* fin and during regeneration. **d** UMAP plots (top) of *hif4a* expression in the uninjured zebrafish caudal fin and during regeneration; violin plots (bottom) of *hif4a* expression across cell types identified in the zebrafish caudal fin regeneration scRNA-seq dataset. Proximal (prox.) distal (dist.) axis indicated in **a.**

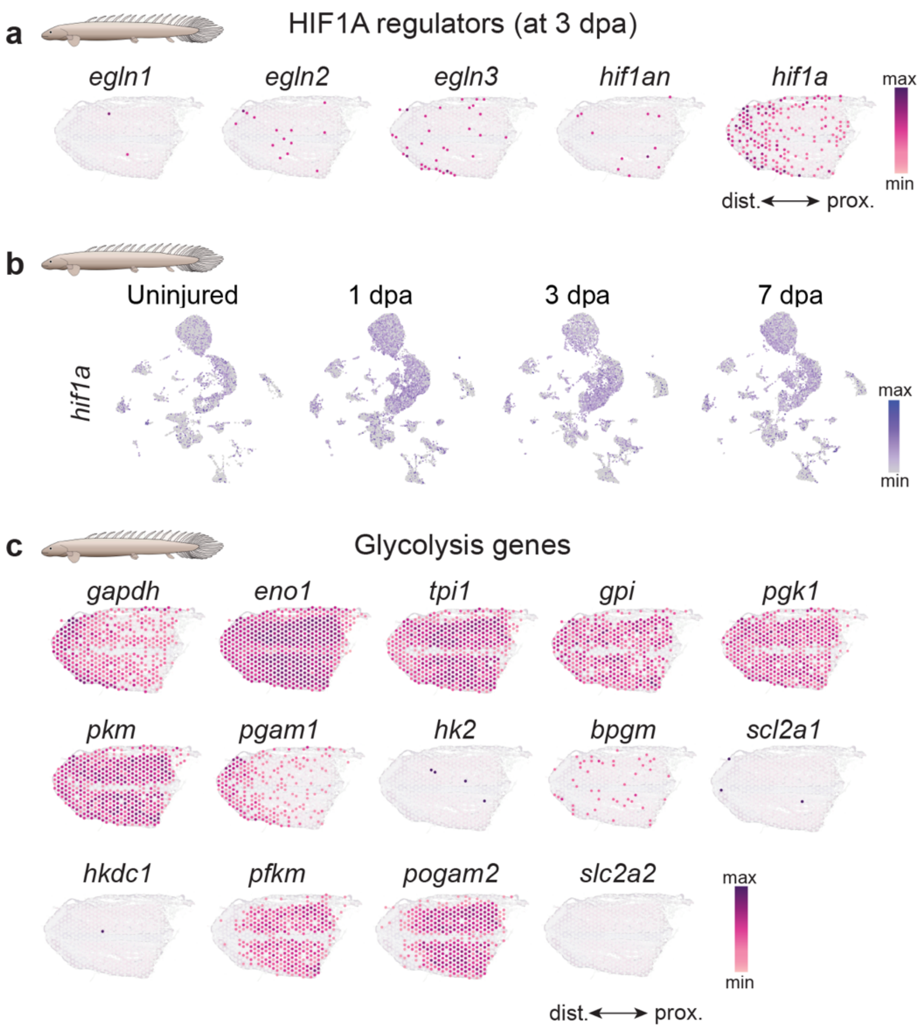

Supplementary Fig. 9. Expression of *hif1a gene*, its regulators, and glycolysis genes during *Polypterus* fin regeneration. Spatial expression patterns of Hif1a regulators at 3 dpa (**a**), UMAP plots of *hif1a* gene expression in the *Polypterus* fin regeneration snRNA-seq dataset (**b**) and spatial RNA-seq expression of glycolysis genes (**c**) at 3 dpa. Proximal (prox.) distal (dist.) axis indicated in **a** and **c**.

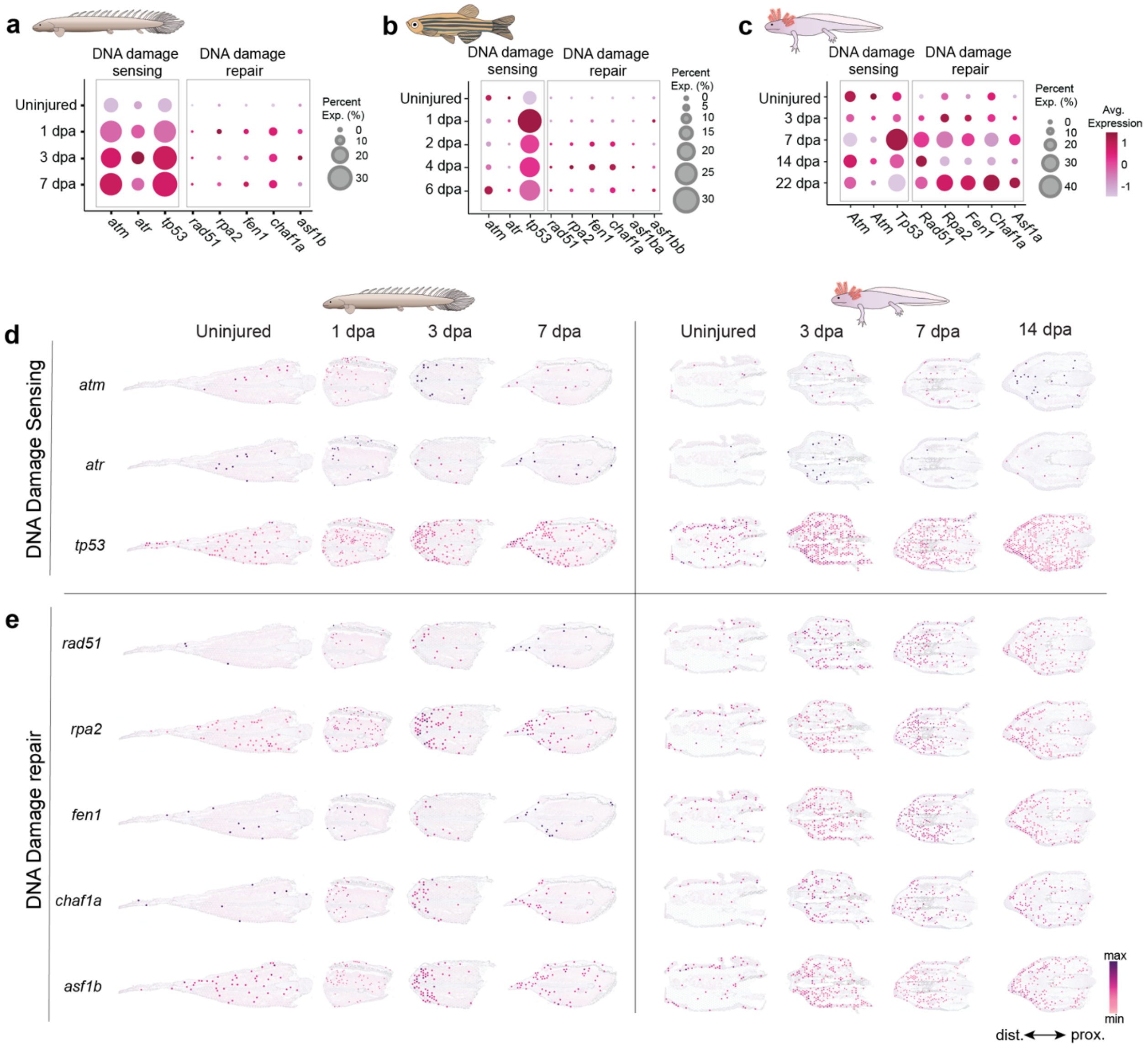

Supplementary Fig. 10. Upregulation of DNA damage sensing and repair markers during limb and fin regeneration. a-c Dot plots of DNA damage sensing and DNA damage repair genes in *Polypterus* (**a**), zebrafish (**b**), and axolotl (**c**). **d** Spatial RNA-seq showing the expression of DNA damage sensing genes in the uninjured *Polypterus* fin and axolotl limb, and during regeneration stages. **e** Spatial RNA-seq showing the expression of DNA damage repair genes in the uninjured *Polypterus* fin and axolotl limb, and during regeneration stages. Proximal (prox.) distal (dist.) axis indicated in **e.**

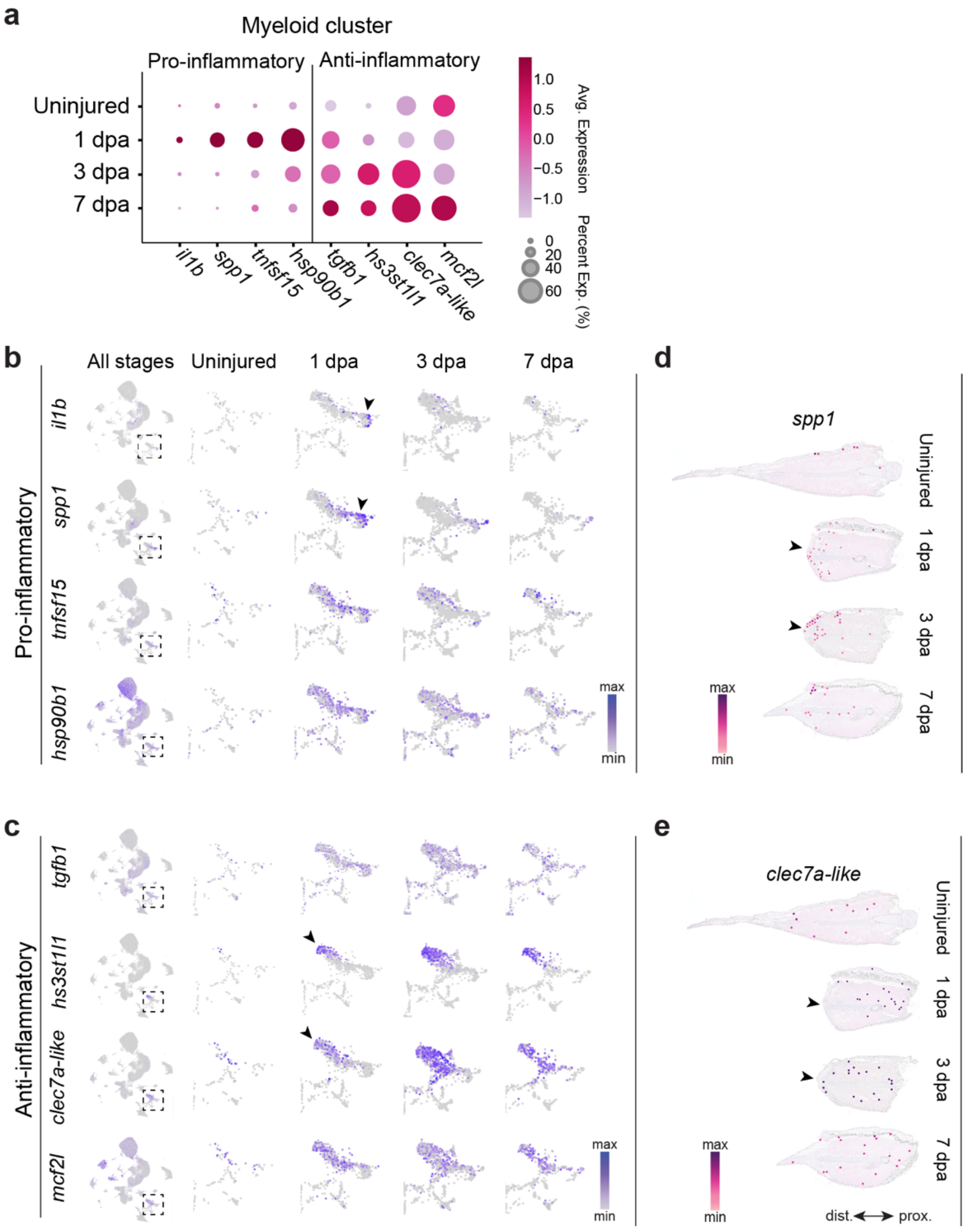

Supplementary Fig. 11. Injury triggers pro- and anti-inflammatory responses during *Polypterus* fin regeneration. a Dot plot of *Polypterus* pro- and anti-inflammatory genes upregulated genes across fin regeneration stages in the myeloid cell cluster. **b, c** UMAP plots showing expression of pro-inflammatory (**b**) and anti-inflammatory (**c**) genes expressed in all stages combined, in the uninjured tissue and across regeneration stages; dashed black boxes zoom in on the myeloid cell cluster; black arrowheads indicate cells within the cluster showing high levels of marker gene expression. **d, e** Spatial RNA-seq showing expression of *spp1* (**d**) and *clec7a-like* (**e**) in the uninjured and regenerating *Polypterus* fin. Black arrowheads (**d, e**) indicate wound epidermis at 1 dpa and 3 dpa. Proximal (prox.) distal (dist.) axis indicated in **e**.

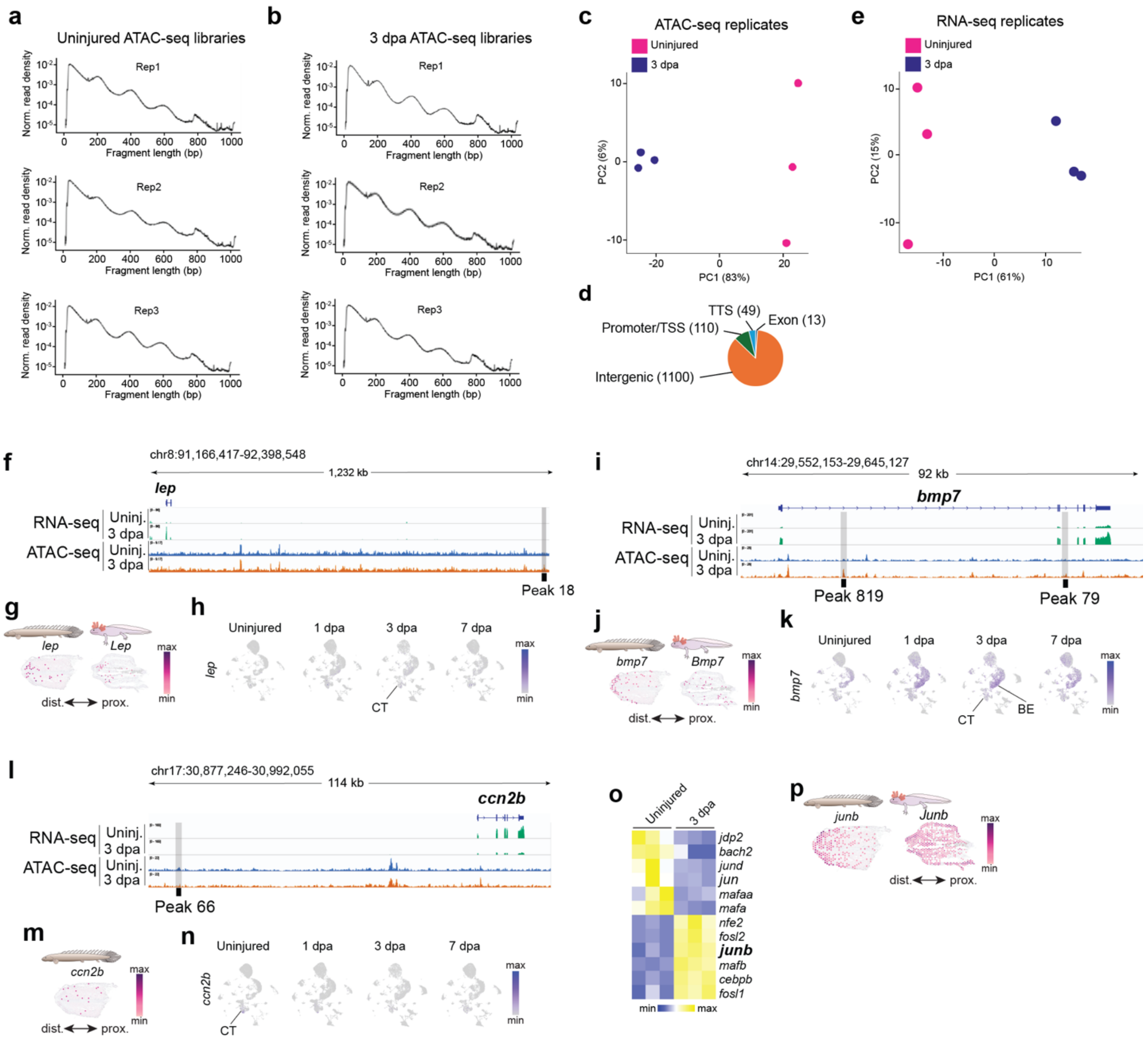

Supplementary Fig. 12. Quality control of bulk ATAC-seq and bulk RNA-seq libraries and additional examples of *Polypterus* candidate TREEs. a, b Fragment distribution plots of ATAC-seq aligned BAM files from uninjured (**a**) and 3 dpa stage (**b**) fin libraries in logarithmic scale. **c** Principal component analysis plots showing separation between ATAC-seq replicates. **d** Annotation of ATAC-seq peaks; number of peaks per category shown in parenthesis. **e** Principal component analysis plots showing separation between RNA-seq replicates. **f, i, l** Genomic location of peak 18 upstream of *lep* (**f**), peaks 79 and 819 upstream of *bmp7* (**i**), and peak 66 upstream of *ccn2b* (**l**)**,** including RNA-seq and ATAC-seq tracks. **g, j, m** Spatial expression of *Polypterus lep*, *bmp7***,** and their respective axolotl orthologs, and *Polypterus ccn2b* at 3 dpa. **h, k, n** *Polypterus* snRNA-seq UMAP plots showing expression of *lep* (**h**), *bmp7* (**k**), and *ccn2b* (**n**). **o** AP-1 TFs differentially expressed in RNA-seq data at 3 dpa relative to uninjured fin tissue. **p** Spatial expression of *Polypterus junb* and its axolotl ortholog. CT, connective tissue; BE, basal epidermis. Proximal (prox.) distal (dist.) axis indicated in **g, j, m** and **p.**
