# Supplementary material for: Comparative multi-omic analysis reveals conserved and derived mechanisms of fin and limb regeneration
Source: Nat Commun. 2026 Jan 22;17:1922. doi: 10.1038/s41467-026-68801-w (PMC12923738; doi:10.1038/s41467-026-68801-w)
Supplement: Supplementary file 3 — Description of Additional Supplementary Files [file 41467_2026_68801_MOESM3_ESM.pdf]

Description of Additional Supplementary Files

File Name: Supplementary Data 1
Description: Top 50 genes differentially expressed in the endoskeleton and fin ray regeneration domains compared to all other clusters, in nuclei from all stages of the *Polypterus* snRNA-seq dataset. Source data for Fig.3.

File Name: Supplementary Data 2
Description: Differentially expressed genes in the basal/intermediate epidermis cluster at 1 dpa, 3 dpa and 7 dpa, relative to the uninjured fin of the *Polypterus* snRNA-seq dataset. Source data for Fig.4 and Supplementary Fig 5.

File Name: Supplementary Data 3
Description: Top 100 genes differentially expressed in the proximal and distal CT clusters compared to all other clusters of the *Polypterus* and axolotl spatial RNA-seq dataset at 7 dpa (*Polypterus*) and 14 dpa (axolotl). Source data for Fig. 7.

File Name: Supplementary Data 4
Description: Top 100 genes differentially expressed in the erythrocyte clusters compared to all other clusters in nuclei or cells from all stages of the *Polypterus* snRNA-seq dataset and axolotl scRNA-seq dataset. Source data for Fig. 8 and Supplementary Fig. 8.

File Name: Supplementary Data 5
Description: Metrics of ATAC-seq read alignment to the *Polypterus* reference genome, and peak calling per condition (uninjured and 3 dpa). Source data for Supplementary Fig. 12a-e

File Name: Supplementary Data 6
Description: Differentially accessible chromatin regions (peaks) between 3 dpa and uninjured *Polypterus* fins. Source data for Fig.10a. Supplementary Fig. 12d.

File Name: Supplementary Data 7
Description: Metrics of bulk RNA-seq read alignment to the *Polypterus* reference genome and annotation to genomic features (uninjured and 3 dpa).

File Name: Supplementary Data 8
Description: Differential gene expression data from *Polypterus* bulk RNA-seq (uninjured and 3 dpa). Source data for Fig.10b and Supplementary Fig. 12o

File Name: Supplementary Data 9
Description: Differential transcription factor footprinting from *Polypterus* ATAC-seq peaks identified as significantly differentially accessible (uninjured and 3 dpa). Source data for Fig.10l.

File Name: Supplementary Data 10
Description: List of manually annotated genes of *Polypterus* and axolotl.
