# Supplementary material for: Comparative multi-omic analysis reveals conserved and derived mechanisms of fin and limb regeneration
Source: Nat Commun. 2026 Jan 22;17:1922. doi: 10.1038/s41467-026-68801-w (PMC12923738; doi:10.1038/s41467-026-68801-w)
Supplement: Supplementary file 14 — Reporting Summary [file 41467_2026_68801_MOESM14_ESM.pdf]

nature portfolio

Corresponding author(s): Igor Schneider

Last updated by author(s): 10/14/2025

Reporting Summary

Nature Portfolio wishes to improve the reproducibility of the work that we publish. This form provides structure for consistency and transparency in reporting. For further information on Nature Portfolio policies, see our Editorial Policies and the Editorial Policy Checklist.

Statistics

For all statistical analyses, confirm that the following items are present in the figure legend, table legend, main text, or Methods section.

n/a	Confirmed	
☐	☒	The exact sample size (*n*) for each experimental group/condition, given as a discrete number and unit of measurement
☐	☒	A statement on whether measurements were taken from distinct samples or whether the same sample was measured repeatedly
☐	☒	The statistical test(s) used AND whether they are one- or two-sided<br>*Only common tests should be described solely by name; describe more complex techniques in the Methods section.*
☒	☐	A description of all covariates tested
☐	☒	A description of any assumptions or corrections, such as tests of normality and adjustment for multiple comparisons
☐	☒	A full description of the statistical parameters including central tendency (e.g. means) or other basic estimates (e.g. regression coefficient) AND variation (e.g. standard deviation) or associated estimates of uncertainty (e.g. confidence intervals)
☐	☒	For null hypothesis testing, the test statistic (e.g. *F*, *t*, *r*) with confidence intervals, effect sizes, degrees of freedom and *P* value noted<br>*Give P values as exact values whenever suitable.*
☒	☐	For Bayesian analysis, information on the choice of priors and Markov chain Monte Carlo settings
☐	☒	For hierarchical and complex designs, identification of the appropriate level for tests and full reporting of outcomes
☒	☐	Estimates of effect sizes (e.g. Cohen's *d*, Pearson's *r*), indicating how they were calculated

Our web collection on statistics for biologists contains articles on many of the points above.

Software and code

Policy information about availability of computer code

Data collection	fastq-dll (v1.0.3)
Data analysis	Data analysis was performed using both commercial and open-source software. Commercial software included Trailmaker (https://app.trailmaker.parsebiosciences.com/v1.4.0, Parse Biosciences, 2024); SpaceRanger 3.1.3; Loupe Browser 8.0.0; CellRanger v7.2.0. Open source software included: STAR v2.7.10b; featureCounts v2.0.6; NGmerge v0.3; Bowtie2 v2.2.5; Samtools v1.18; Genrich v0.6.1; IGV v 2.14.1; HOMER v 4.11; TOBIAS v0.17.0; R packages including Seurat v4.4.1, DESeq2 v1.40.2, ATACseqQC v1.24.0. Custom codes are available in: https://github.com/lnperez90/Sousa-et-al-2025.

For manuscripts utilizing custom algorithms or software that are central to the research but not yet described in published literature, software must be made available to editors and reviewers. We strongly encourage code deposition in a community repository (e.g. GitHub). See the Nature Portfolio guidelines for submitting code & software for further information.

Data

Policy information about availability of data

All manuscripts must include a data availability statement. This statement should provide the following information, where applicable:

- Accession codes, unique identifiers, or web links for publicly available datasets
- A description of any restrictions on data availability
- For clinical datasets or third party data, please ensure that the statement adheres to our policy

All raw and processed data from bulk RNA-seq, spatial transcriptomics, snRNA-seq and bulk ATAC-seq has been deposited in the Gene Expression Omnibus (GEO)

under the accession numbers GSE XXX, GSE XXX, GSEXXX and GSEXXX, respectively. Processed Polypterus snRNA-seq data have been made available at the Broad Single Cell Portal (https://singlecell.broadinstitute.org/single_cell) (SCP: SCP3138). SnRNA-seq data from zebrafish were obtained from the Gene Expression Omnibus (GEO; accession number GSE261907). The axolotl scRNA-seq dataset was downloaded from the NCBI Sequence Read Archive (SRA; project number PRJNA589484).

Research involving human participants, their data, or biological material

Policy information about studies with human participants or human data. See also policy information about sex, gender (identity/presentation), and sexual orientation and race, ethnicity and racism.

Reporting on sex and gender	NA. This study did not involve human participants or human data.
Reporting on race, ethnicity, or other socially relevant groupings	NA. This study did not involve human participants or human data.
Population characteristics	NA. This study did not involve human participants or human data.
Recruitment	NA. This study did not involve human participants or human data.
Ethics oversight	NA. This study did not involve human participants or human data.

Note that full information on the approval of the study protocol must also be provided in the manuscript.

Field-specific reporting

Please select the one below that is the best fit for your research. If you are not sure, read the appropriate sections before making your selection.

☒ Life sciences ☐ Behavioural & social sciences ☐ Ecological, evolutionary & environmental sciences

For a reference copy of the document with all sections, see nature.com/documents/nr-reporting-summary-flat.pdf

Life sciences study design

All studies must disclose on these points even when the disclosure is negative.

Sample size	No statistical method was used to determine sample size. The number of biological replicates for bulk, single-nucleus, and spatial transcriptomics experiments was based on feasibility and consistency with previously published studies in Polypterus, zebrafish, and axolotl regeneration models.
Data exclusions	Low quality nuclei from the the snRNA-seq experiment were excluded during data processing using the Trailmaker software (Parse Biosicences) as described in the Methods section.
Replication	Three biological replicates were used for both bulk RNA-seq and bulk ATAC-seq experiments on fins at homeostasis (uninjured) and at 3 days post-amputation (dpa). For snRNA-seq, two biological replicates—each consisting of pooled samples from two animals—were prepared for each time point. Spatial transcriptomics was performed on single tissue sections from each of the four time points analyzed for Polypterus fins (uninjured, 1, 3, and 7 dpa) and axolotl limbs (uninjured, 3, 7, and 14 dpa). Histology images from Polypterus intact and regenerating fins were selected from section series obtained from a pair of pectoral fins from two animals per stage. HCR-FISH analyses were conducted using at least two technical replicates for each of the four Polypterus fin time points (uninjured, 1, 3, and 7 dpa).
Randomization	Animals within a defined size range (Polypterus 7–10 cm; axolotl 8–12 cm) were randomly assigned to groups for collection of regenerating fins or limbs at different time points.
Blinding	Blinding was not applied in this study. Experimental sample collection and molecular assays followed standardized protocols, and data analyses were conducted using automated bioinformatics pipelines, reducing the potential for bias.

Reporting for specific materials, systems and methods

We require information from authors about some types of materials, experimental systems and methods used in many studies. Here, indicate whether each material, system or method listed is relevant to your study. If you are not sure if a list item applies to your research, read the appropriate section before selecting a response.

Materials & experimental systems

n/a	Involved in the study
☒	☐ Antibodies
☒	☐ Eukaryotic cell lines
☒	☐ Palaeontology and archaeology
☐	☒ Animals and other organisms
☒	☐ Clinical data
☒	☐ Dual use research of concern
☒	☐ Plants

Methods

n/a	Involved in the study
☒	☐ ChIP-seq
☒	☐ Flow cytometry
☒	☐ MRI-based neuroimaging

Animals and other research organisms

Policy information about studies involving animals; ARRIVE guidelines recommended for reporting animal research, and Sex and Gender in Research

Laboratory animals	Polypterus senegalus (obtained from pet suppliers), Ambystoma mexicanum (Ambystoma Stock Center, Lexington, Kentucky), All animals were juveniles (exact age undetermined).
Wild animals	The study did not involve wild animals
Reporting on sex	Given the absence of distinguishing sexual traits, sex was not considered in this study
Field-collected samples	The study did not involve field collections.
Ethics oversight	Animals were maintained and used in accordance with an approved Louisiana State University (LSU) IACUC protocol IACUCAM-25-047.

Note that full information on the approval of the study protocol must also be provided in the manuscript.

Plants

Seed stocks	*Report on the source of all seed stocks or other plant material used. If applicable, state the seed stock centre and catalogue number. If plant specimens were collected from the field, describe the collection location, date and sampling procedures.*
Novel plant genotypes	*Describe the methods by which all novel plant genotypes were produced. This includes those generated by transgenic approaches, gene editing, chemical/radiation-based mutagenesis and hybridization. For transgenic lines, describe the transformation method, the number of independent lines analyzed and the generation upon which experiments were performed. For gene-edited lines, describe the editor used, the endogenous sequence targeted for editing, the targeting guide RNA sequence (if applicable) and how the editor was applied.*
Authentication	*Describe any authentication procedures for each seed stock used or novel genotype generated. Describe any experiments used to assess the effect of a mutation and, where applicable, how potential secondary effects (e.g. second site T-DNA insertions, mosiacism, off-target gene editing) were examined.*
